# Outbreak of Norovirus GII.P17-GII.17 in the Canadian Province of Nova Scotia

**DOI:** 10.1155/2016/1280247

**Published:** 2016-05-08

**Authors:** Jason J. LeBlanc, Janice Pettipas, Daniel Gaston, Robin Taylor, Todd F. Hatchette, Tim F. Booth, Russell Mandes, Andrew McDermid, Elsie Grudeski

**Affiliations:** ^1^Nova Scotia Health Authority (NSHA), Halifax, NS, Canada B3H 1V8; ^2^Dalhousie University, Halifax, NS, Canada B3H 4R2; ^3^Provincial Public Health Laboratory Network of Nova Scotia (PPHLN), Halifax, NS, Canada B3H 1V8; ^4^National Microbiology Laboratory (NML), Winnipeg, MB, Canada R3E 3R2; ^5^University of Manitoba, Winnipeg, MB, Canada R3T 2N2

## Abstract

*Background*. Norovirus is the leading cause of viral gastroenteritis, with GII.4 being the most common circulating genotype. Recently, outbreaks in China revealed that norovirus GII.17 GII.P17 had become predominant.* Objective*. This study aimed to characterize the distribution of norovirus genotypes circulating in Nova Scotia.* Methods*. Stool specimens were collected from gastrointestinal outbreaks in Nova Scotia between Jan 2014 and June 2015 and subjected to real-time RT-PCR. Norovirus-positive specimens were referred to the National Microbiology Laboratory for sequence-based genotyping.* Results*. The first norovirus GII.P17-GII.17 outbreak in Canada was identified, but no widespread activity was observed in Nova Scotia.* Discussion*. It is unknown whether GII.P17-GII.17 is more widespread in Canada since contributions to Canadian surveillance are too sparse to effectively monitor the epidemiology of emerging norovirus genotypes.* Conclusions*. Presence of norovirus GII.17:P17 in Canada highlights the need for more systematic surveillance to ensure that molecular targets used for laboratory detection are effective and help understand norovirus evolution, epidemiology, and pathogenesis.

## 1. Introduction

Norovirus is the leading cause of viral gastroenteritis, and outbreaks are common [1]. Transmission can be reduced through infection control and public health interventions, and laboratory identification is helpful [1]. The method of choice for norovirus detection is reverse-transcription polymerase chain reaction (RT-PCR); however, the genetic diversity among noroviruses and emergence of novel variants can be challenging [2, 3]. As such, monitoring the epidemiology of circulating noroviruses is important to ensure accurate detection in clinical laboratories.

Human noroviruses are single stranded positive sense RNA comprised of three open reading frames (ORF 1 to 3), encoding RNA-dependent RNA polymerase (RdRp), major viral capsid protein (VP1), and minor capsid protein (VP2), respectively [2]. Based on amino acid differences in VP1, noroviruses are classified into six genogroups (GI to GVI), which are further subdivided into 9, 22, and 2 genotypes, respectively [2, 4]. Only 9 genotypes of GI, 19 genotypes of GII, and only one genotype of GIV are known to cause human disease [2, 4]. Each year, a number of norovirus genotypes circulate with GII.4 being predominant, and novel GII.4 variants arise every two to three years to replace the previously circulating pandemic strain [5, 6]. Recently, outbreaks in China revealed that a GII.17 variant (GII.P17-GII.17) had become predominant, replacing pandemic GII.4 [7–9]. The potential for widespread circulation of genotype GII.P17-GII.17 has led to recent interest [7–10]. Here we describe the first cases of norovirus GII.P17-GII.17 in Canada, termed GII/Hu/CA/2014/GII.P17-GII.17/NS_063 and GII/Hu/CA/2014/GII.P17-GII.17/NS_065.

## 2. Methods

Stool specimens were collected as part of routine acute gastroenteritis outbreaks investigations in Nova Scotia from Jan. 2014 to June 2015. Each specimen submitted from outbreak investigation was tested for norovirus using real-time RT-PCR, and one to three norovirus-positive specimens from outbreaks were submitted for genotypic analyses at the National Microbiology Laboratory (NML) in Winnipeg, MB.

### 2.1. Gastroenteritis Outbreak Investigation

Public healthoutbreak investigation data was provided by the Nova Scotia Department of Health and Wellness (Halifax, NS). The clinical case definition for gastroenteritis outbreak was defined as follows: two or more loose watery stools (above normal) or two or more episodes of vomiting or both, occurring within a 24-hour period, or identification of a pathogen and at least one gastrointestinal symptom. Stool specimens were submitted for norovirus, rotavirus, and adenovirus testing at the Division of Microbiology, Queen Elizabeth II Health Sciences Centre, Halifax, NS.

### 2.2. Specimen Preparation

Rotavirus and adenovirus testing was performed on fresh stool using the Rota/Adenoscreen Cassette (Microgen Bioproducts), as recommended by the manufacturer. For norovirus testing, stool slurries were prepared by transferring 200 *μ*L of stool into 500 *μ*L of PCR-grade water and centrifugation (10,000 ×g, 10 min). The supernatants (140 *μ*L) were subjected to a total nucleic acid (TNA) extraction on a MagNA Pure LC instrument (Roche Diagnostics, Branchburg, NJ), as recommended by the manufacturer. TNAs were eluted in a volume of 60 *μ*L and 5 *μ*L served as template for norovirus real-time RT-PCR. Primers and probes were synthesized by Sigma Genosys (Oakville, ON) (Table S1 in Supplementary Material available online at http://dx.doi.org/10.1155/2016/1280247).

### 2.3. Norovirus Real-Time RT-PCR

Real-time RT-PCR was performed in a duplex reaction by combining primers and probes commonly used for GI ad GII noroviruses [3]. Briefly, PCR amplifications were performed on a Life Technologies ABI 7500 Fast instrument in 25 *μ*L reactions consisting of the following: SuperScript III Platinum One-Step 1x master mix (Life Technologies), 0.2 *μ*L enzyme mix, 20 U RNaseOUT, and 400 nM of each primer and probe (Table S1). Amplification conditions were as follows: 50°C for 30 min; 95°C for 30 s; and 45 cycles of 95°C for 30 s and 60°C for 1 min. Ct values were determined using the manufacturer's software (version 2.0.5).

### 2.4. Norovirus Genotyping and Sequence Analyses

For genotyping, RT-PCR amplification and sequencing were performed for both polymerase and capsid genes. Briefly, RNA was transcribed into cDNA using SuperScript Vilo master mix (Invitrogen) according to manufacturer's instructions. Regions B/C and D RT-PCR reactions were performed using a Platinum Taq polymerase (Invitrogen) in 50 *μ*L reactions consisting of the following: 0.5 *μ*L enzyme, 1x buffer, 400 nM dNTPs, 1.5 mM MgCl_2_, 10 *μ*L of cDNA, and 500 nM of each primer (431F and G2SKR for regions B and C and primers CapCRev, CapD3Fwd, and CapD1 Fwd) (Table S1). Amplification conditions were as follows: 94°C for 6 min; 40 cycles of 94°C for 30 s, 40°C (region D) or 50°C (region B/C) for 30 s, and 72°C for 30 s; and a final extension of 72°C for 5 min. Following 2% agarose gel electrophoresis, the amplicons were purified using Amicon Filter Devices (Millipore, USA) and sequencing was carried out by the Genomics Core section of the NML (Figure S1). For polymerase genotype (GII.P17) assignment, the sequences were submitted to the Norovirus Automated Genotyping Tool reference dataset [11].

To further characterize the Nova Scotia GII.P17 strain, the full VP1 capsid gene (ORF 2) was sequenced and phylogenetic analysis was used to assign the capsid genotype (GII.17). Briefly, ORF 2 was amplified using SuperScript III One-Step RT-PCR System with Platinum* Taq* High Fidelity DNA Polymerase Kit in a 50 *μ*L reaction consisting of 25 *μ*L 2x reaction mix, 1 *μ*L enzyme mix, 1.2 *μ*M of each primer (COG2F and GV132R, Table S1), and 10 *μ*L of template. Thermocycling conditions were as follows: 50°C for 30 min; 94°C for 2 min; 45 cycles of 94°C for 30 s and 50°C for 30 s; 68°C for 2 min; and a final extension of 68°C for 7 min. Following 1% agarose gel electrophoresis, amplicons were purified and sequenced using primers listed in Table S1.

### 2.5. Phylogenetic Analyses

**Figure 1:**
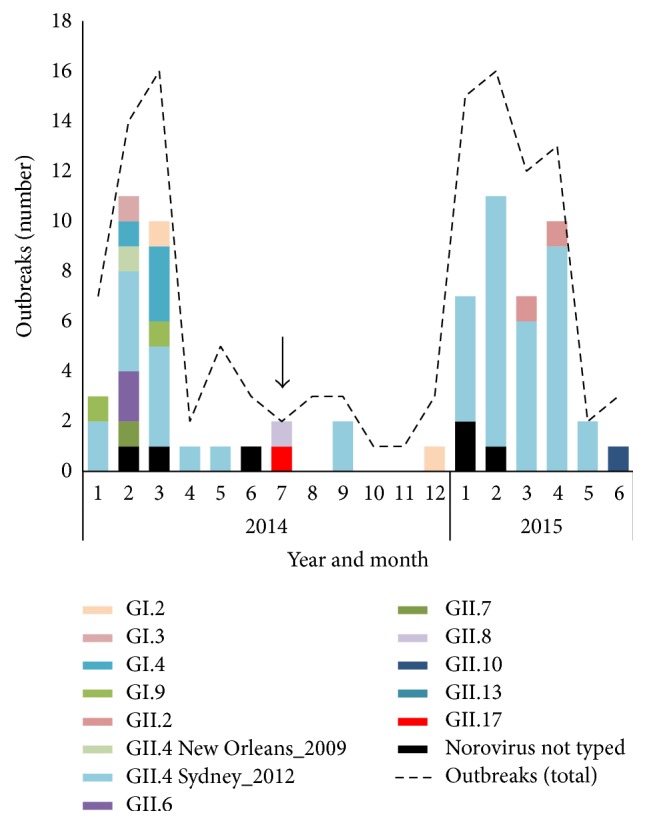
Distribution of norovirus genotype in Nova Scotia from January 2014 to June 2015. The total number of viral enteric outbreaks is indicated by a dashed line. The arrow represents the appearance of norovirus genotype GII.P17-GII.17.

Sequence data was analyzed using Sequencher version 5.2.4 (GeneCodes Corp., Ann Arbor, MI). BioNumerics 5.1 software (Applied Maths, Austin, TX) was used to assemble consensus sequence data. Multiple sequence alignments were generated with BioEdit version 7.2.5. Maximum-likelihood estimation of the phylogenetic tree used the General Time Reversible (GTR) model of sequence substitution with 4 gamma-distributed rate categories. Phylogenetic trees were constructed using RAxML (version 8) and combined rapid bootstrapping and maximum-likelihood search algorithm (-f a) with 1000 bootstrap replicates, followed by ML refinement (-f T). Phylogenetic analysis was performed using the CIPRES science gateway [12]. Trees were visualized with EvolView [13]. To compare primer/probe binding sites to the target sequences of circulating norovirus genotypes (Figure 1), sequence data was retrieved from the Genbank database on the NCBI website [14] and pairwise sequence alignments were performed using the BLAST function [15].

## 3. Results

### 3.1. Gastroenteritis Outbreaks

Of the 121 gastroenteritis outbreaks declared in Nova Scotia from January 2014 to June 2015, 18 were reported from acute care facilities and 103 from long term care facilities. Twenty-five (20.7%) outbreaks were declared based on clinical presentation alone. Of the 96 outbreaks where specimens had been submitted for laboratory testing, norovirus was detected in 70 (72.9%), rotavirus in three (3.1%), and adenovirus in one (1.0%). For norovirus, a genotype was assigned in 64 of the 70 outbreaks, with outbreaks predominantly caused by GII.4 at 73.4% (47/64), and infrequent reports of other genotypes (Figure 1). The distribution of norovirus genotypes remained relatively unchanged one year following the appearance of norovirus GII.P17-GII.17 in Nova Scotia (Figure 1).

### 3.2. Outbreak of Norovirus GII.P17-GII.17

The outbreak identified as being caused by norovirus GII.P17-GII.17 began July 15, 2014, in a long term care facility in the central zone of the Nova Scotia Health Authority (NSHA). Fourteen residents met the clinical case definition for gastroenteritis outbreak. No staff members were tested; however, 10 declared symptoms during the outbreak. Of the four residents tested using norovirus real-time RT-PCR [3], three had noroviruses identified in their stool (with threshold cycle values of 18.18, 20.52, and 32.39). The NML confirmed the presence of norovirus in two of the three specimens, and genotype was assigned as GII.P17-GII.17 using a dual-target nomenclature based on polymerase and capsid sequences [4]. How norovirus GII.P17-GII.17 was introduced in Nova Scotia is not clear, as there was no evidence of travel in any affected individual. Following public health interventions, the outbreak was declared over on July 30.

### 3.3. Phylogenetic Analyses

**Figure 2:**
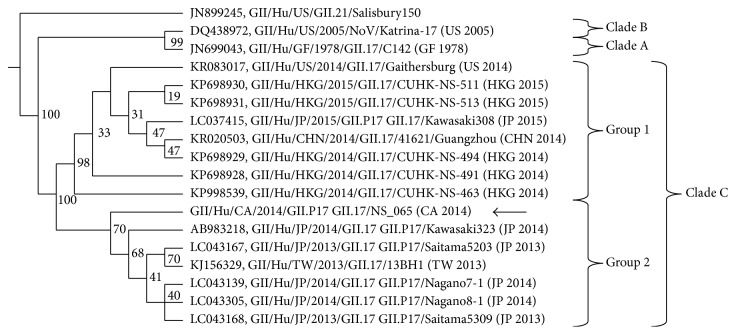
Phylogenetic analysis of the full length VP1 nucleotide sequences of various noroviruses. Presented is a cladogram with supporting bootstrap values. Arrows indicating the location of the Nova Scotia sequences.

**Figure 3:**
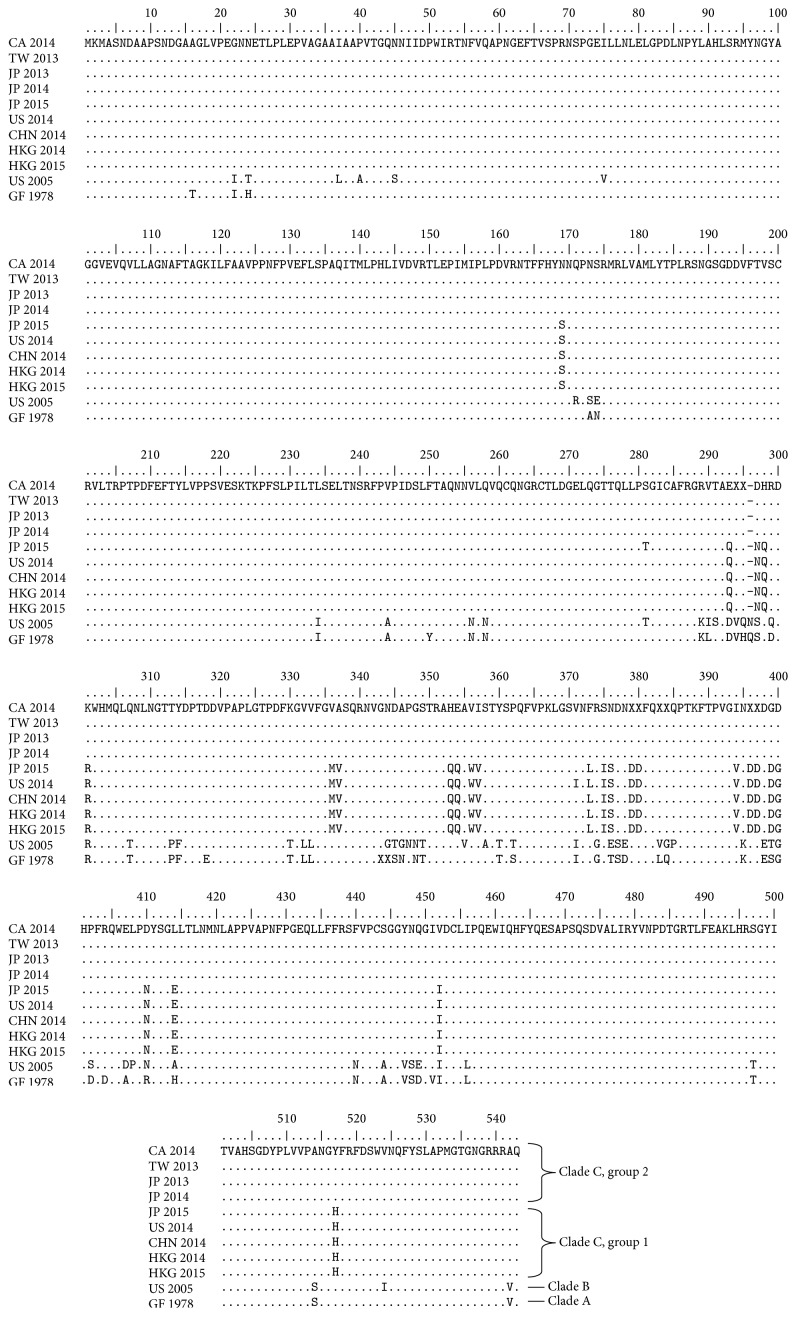
Multiple sequence alignment comparing the full length VP1 sequences of GII.P17-GII.17 noroviruses. Alignments were generated with BioEdit version 7.2.5. Genbank accession numbers, strain nomenclature, and abbreviations used in the figure are as follows: GII/Hu/CA/2014/GII.P17 GII.17/NS_065 (CA 2014); KJ156329, GII/Hu/TW/2013/GII.17/13BH1 (TW 2013); LC043168, GII/Hu/JP/2013/GII.17 GII.P17/Saitama5309 (JP 2013); AB983218, GII/Hu/JP/2014/GII.17 GII.P17/Kawasaki323 (JP 2014); KR083017, GII/Hu/US/2014/GII.17/Gaithersburg (US 2014); KR020503, GII/Hu/CHN/2014/GII.17/41621/Guangzhou (CHN 2014); KP998539, GII/Hu/HKG/2014/GII.17/CUHK-NS-463 (HKG 2014); KP698931, GII/Hu/HKG/2015/GII.17/CUHK-NS-513 (HKG 2015); LC037415, GII/Hu/JP/2015/GII.P17 GII.17/Kawasaki308 (JP 2015); DQ438972, GII/Hu/US/2005/NoV/Katrina-17 (US 2005); JN699043, GII/Hu/GF/1978/GII.17/C142 (GF 1978).

The two noroviruses sequenced by the NML revealed identical sequences for all targets in both specimens. Sequencing of regions B and C of the ORF 1 and 2 yielded a 535 bp sequence (Figure S1) that was identified as polymerase genotype GII.P17 [11]. The sequence clustered to recent lineages of GII.P17-GII.17 in Japan, China, and US (Figures 2 and 3) [7–9]. Next, phylogenetic analysis of the full length VP1 sequence was used to assign the capsid genotype (GII.17). Once again, the sequences clustered to clade C of recent GII.P17-GII.17 noroviruses [9] and diverged from the historical GII.17 clades A and B (Figures 2 and 3). Interestingly, the recent GII.P17-GII.17 noroviruses in clade C could be divided into two groups based on nucleotide and amino acid composition in major surface epitopes (Figures 2 and 3).

## 4. Discussion

Norovirus GII.P17-GII.17 first emerged in October 2014 as the predominant genotype of norovirus causing acute gastroenteritis in China. The experts have forecasted that this novel genotype will become the next global epidemic [7–9], highlighting the importance of tracking outbreaks caused by this strain globally. This study provides the first report of presence of norovirus GII.P17-GII.17 in Canada. However, unlike the rapid emergence and replacement of the pandemic GII.4 strain by GII.P17-GII.17 in China and Japan [7–9], the distribution of norovirus genotypes remained relatively unchanged one year following its appearance in Nova Scotia (Figure 2). No further outbreaks of this genotype have been declared in Canada, and only sporadic cases have been seen in other countries [8, 9].

Despite the lack of widespread GII.P17-GII.17 activity in Nova Scotia, its appearance in July 2014 is of interest since the outbreak predates the timeline of reports from China and US (in the winter months of 2014-2015). Noroviruses are thought to emerge in human populations through antigenic drift genetic recombination or mutation in surface proteins like VP1, leading to evasion of preexisting herd immunity in the human population [6, 7]. As such, further characterization of its genome may be of interest to help understand norovirus evolution, pathogenesis, and spread [7–9]. The Nova Scotia sequences were identical to each other and to GII.P17-GII.17 noroviruses circulating in Japan and Taiwan in 2013 and 2014 (Figure S2). However, compared to recent GII.17 noroviruses in China and Japan in 2014 and 2015, several amino substitutions were noted including some positioned in major epitopes [9], which could explain differences in infectivity and spread (Figure 3). Other recent studies have supported the observation that there are genetic differences between GII.17 noroviruses and suggested this virus may be undergoing genetic diversification [16, 17]. Further genetic characterization is underway.

The lack of further detection of GII.P17-GII.17 in Nova Scotia was not thought to be attributed to the detection method, since norovirus GII.P17-GII.17 was readily detected using a real-time RT-PCR commonly used in many Canadian laboratories, and in silico analyses did not show any target site mutations (Figure S2). It would be interesting to compare the performance of commercial and in-house molecular assays for the detection of norovirus GII.P17-GII.17 since low sensitivity was recently reported for immunochromatographic methods [10]. It is also possible that some additional outbreaks of norovirus GII.P17-GII.17 might have gone unnoticed with suboptimal number (or complete lack) of specimens submitted for laboratory detection and lack of routine norovirus genotyping in most Canadian laboratories. Unlike other surveillance networks [2, 18], contributions to Canadian surveillance are too sparse to effectively monitor the epidemiology of emerging norovirus genotypes and as such it is unknown whether norovirus GII.P17-GII.17 is more widespread.

Overall, this paper reports the first outbreak in Canada of a novel norovirus genotype GII.P17-GII.17. While this novel norovirus may be a harbinger of change in norovirus epidemiology, no further reports have been described in Canada. Whether norovirus GII.P17-GII.17 will eventually displace the current pandemic GII.4 strain is unknown; however, presence in Canada highlights the need for more systematic surveillance to ensure that molecular targets used for laboratory detection are effective and help understand the norovirus evolution, epidemiology, and pathogenesis.

## Supplementary Material

To identify norovirus GII.P17-GII.17, a dual target nomenclature was used based on sequences for the viral polymerase (Figure S1) and VP1 capsid (Figure 2). Figure S1 represents an alignment of the viral polymerase sequence of norovirus GII.P17-GII17 and related noroviruses. Figure S2 is an alignment of the GIIP17.GII.17 region corresponding to the primers and probes used for real-time RT-PCR detection in many Canadian Public Health Laboratories. Table S1 is a list of the primers and probes used in this study.
