# Supplementary material for: Outbreak of Norovirus GII.P17-GII.17 in the Canadian Province of Nova Scotia
Source: Can J Infect Dis Med Microbiol. 2016 May 8;2016:1280247. doi: 10.1155/2016/1280247 (PMC4904589; doi:10.1155/2016/1280247)
Supplement: Supplementary file 1 — To identify norovirus GII.P17-GII.17, a dual target nomenclature was used based on sequences for the viral polymerase (Figure S1) and VP1 capsid (Figure 2). Figure S1 represents an alignment of the viral polymerase sequence of norovirus GII.P17-GII17 and related noroviruses. Figure S2 is an alignment of the GIIP17.GII.17 region corresponding to the primers and probes used for real-time RT-PCR detection in many Canadian Public Health Laboratories. Table S1 is a list of the primers and probes used in this study. [file 1280247.f1.pdf]

SUPPLEMENTAL INFORMATION

Figure legends

Figure S1. DNA sequence for region B and C of norovirus GII.P17-GII.17 in Nova Scotia.

Figure S2. Real-time RT-PCR primers and probe targets compared to various GII.17 noroviruses. Primer pairs COG2F and COG2R and RING2 were compared to norovirus GII.17 sequences retrieved from the Genbank database and pairwise sequence alignments were performed using BLAST analysis. Genbank accession numbers, strain nomenclature, and abbreviations used in the figure are as follows: KC597139, GII/Hu/GF/1978/GII.17/C142 (GF 1978); EF529741, GII/Hu/FR/2004/GII.17/Briancon870 (FR 2004); AB447409, GII/Hu/IND/2006/GII.17/1668 06 (IND 2006); JQ751044, GII/Hu/CHN/2007/GII.17/Wuhan Z776 (CHN 2007); JF802507, GII/Hu/AF/2009/GII.17/Cameroon C15b (AF 2009); KR020503, GII/Hu/CHN/2014/GII.17/Guangzhou 41621 (CHN 2014); AB983218, GII/Hu/JP/2014/GII.P17 GII.17/Kawasaki323 (JP 2014); KR083017, GII/Hu/US/2014/GII.17/Gaithersburg (US 2014), and GII/Hu/CA/2014/GII.P17 GII.17/NS_065 (CA 2014). Nucleotide abbreviations include: B = C, G, or T; R = A or G; Y = C or T; N = A, C, G, or T.

SUPPLEMENTAL INFORMATION

```
....|....| ....|....| ....|....| ....|....| ....|....| ....|....|
        5         15         25         35         45         55
TGAAGATCCC AGTGAGACCA TGATACCACA TGCACAGAGA CCTGTGCAGC TCATGGCACT

....|....| ....|....| ....|....| ....|....| ....|....| ....|....|
       65         75         85         95        105        115
ACTAGGAGAA TCCTCCCTAC ATGGACCCTC ATTTTACAGC AAGGTTAGCA AACTGGTCAT

....|....| ....|....| ....|....| ....|....| ....|....| ....|....|
      125        135        145        155        165        175
ATCTGAACTT AAAGAGGGAG GAATGGATTT TTATGTGCCC AGACAAGAGT CAATGTTCAG

....|....| ....|....| ....|....| ....|....| ....|....| ....|....|
      185        195        205        215        225        235
ATGGATGAGG TTCTCAGATC TAAGCACATG GGAGGGCGAT CGCAATCTGG CTCCCAGTTT

....|....| ....|....| ....|....| ....|....| ....|....| ....|....|
      245        255        265        275        285        295
TGTGAATGAA GATGGCGTCG AATGACGCCG CTCCATCTAA TGATGGTGCT GCTGGTCTCG

....|....| ....|....| ....|....| ....|....| ....|....| ....|....|
      305        315        325        335        345        355
TACCAGAGGG CAATAACGAG ACCCTTCCCC TAGAACCAGT TGCGGGCGCA GCTATAGCCG

....|....| ....|....| ....|....| ....|....| ....|....| ....|....|
      365        375        385        395        405        415
CACCCGTCAC TGGCCAAAAC AACATAATTG ACCCCTGGAT TAGAACAAAT TTTGTGCAAG

....|....| ....|....| ....|....| ....|....| ....|....| ....|....|
      425        435        445        455        465        475
CACCAAATGG GGAGTTCACA GTGTCACCCA GAAACTCCCC TGGAGAAATT TTACTAAACC

....|....| ....|....| ....|....| ....|....| ....|....| ....|
      485        495        505        515        525        535
TAGAGTTGGG CCCTGATTTG AACCCTTACT TGGCTCATTT GTCAAGGATG TACAA
```

Figure S1. DNA sequence for region B and C of norovirus GII.P17-GII.17 in Nova Scotia.

SUPPLEMENTAL INFORMATION

```
COG2F      CARGARBCNATGTTYAGRTGGATGAG   --------------------   ---------------------
RING2      --------------------------   TGGGAGGGCGATCGCAATCT   ---------------------
COG2R      --------------------------   --------------------   TGTGAATGAAGATGGCGTCGA

GF 1978    CAAGAGCCAATGTTCAGATGGATGAG   TGGGAGGGCGATCGCAATCT   TGTGAATGATGATGGCGTCGA

FR 2004    CAAGAGTCAATGTTCAGATGGATGAG   TGGGAGGGCGATCGCAATCT   TGTGAATGAAGATGGCGTCGA
IND 2006   CAAGAGTCAATGTTCAGATGGATGAG   TGGGAGGGCGATCGCAATCT   TGTGAATGAAGATGGCGTCGA
CHN 2007   CAAGAGTCAATGTTCAGATGGATGAG   TGGGAGGGCGATCGCAATCT   TGTGAATGAAGATGGCGTCGA
AF  2009   CAAGAGTCAATGTTCAGATGGATGAG   TGGGAGGGCGATCGCAATCT   TGTGAATGAAGATGGCGTCGA

CHN 2014   CAAGAGTCAATGTTCAGATGGATGAG   TGGGAGGGCGATCGCAATCT   TGTGAATGAAGATGGCGTCGA
JP  2014   CAAGAGTCAATGTTCAGATGGATGAG   TGGGAGGGCGATCGCAATCT   TGTGAATGAAGATGGCGTCGA
US  2014   CAAGAGTCAATGTTCAGATGGATGAG   TGGGAGGGCGATCGCAATCT   TGTGAATGAAGATGGCGTCGA
CA  2014   CAAGAGTCAATGTTCAGATGGATGAG   TGGGAGGGCGATCGCAATCT   TGTGAATGAAGATGGCGTCGA
```

Figure S2. Real-time RT-PCR primers and probe targets compared to various GII.17 noroviruses. Primer pairs COG2F and COG2R and RING2 were compared to norovirus GII.17 sequences retrieved from the Genbank database and pairwise sequence alignments were performed using BLAST analysis. Genbank accession numbers, strain nomenclature, and abbreviations used in the figure are as follows: KC597139, GII/Hu/GF/1978/GII.17/C142 (GF 1978); EF529741, GII/Hu/FR/2004/GII.17/Briancon870 (FR 2004); AB447409, GII/Hu/IND/2006/GII.17/1668 06 (IND 2006); JQ751044, GII/Hu/CHN/2007/GII.17/Wuhan Z776 (CHN 2007); JF802507, GII/Hu/AF/2009/GII.17/Cameroon C15b (AF 2009); KR020503, GII/Hu/CHN/2014/GII.17/Guangzhou 41621 (CHN 2014); AB983218, GII/Hu/JP/2014/GII.P17 GII.17/Kawasaki323 (JP 2014); KR083017, GII/Hu/US/2014/GII.17/Gaithersburg (US 2014), and GII/Hu/CA/2014/GII.P17 GII.17/NS_065 (CA 2014). Nucleotide abbreviations include: B = C, G, or T; R = A or G; Y = C or T; N = A, C, G, or T.

SUPPLEMENTAL INFORMATION

Table S1. Primer and probes used in this study.

Name	Sequence (5’-3’)	Location*	Target (amplicon size)	Ref.
COG2F	CARGARBCNATGTTYAGRTGGATGAG	4997-5002	ORF1-2 junction (77 bp)	(*19*)
COG2R	TCGACGCCATCTTCATTCACA	5054-5074		(*19*)
Ring2	FAM -TGGGAGGGCGATCGCAATCT-BHQ1	5022-5041		(*20*)
431F	TGGACIAGRGGICCYAAYCA	4794-4813	region B and C (570 bp)	(*21*)
G2SKR	CCRCCNGCATRHCCRTTRTACAT	5341-5363		(*22*)
CapC Rev	CCTTYCCAKWTCCCAYGG	6644-6662	ORF2 region D (253 bp)	(*22*)
CapD3 Fwd	TGYCTYITICCHCARGAATGG	6409-6429		(*22*)
CapD1 Fwd	TGTCTRSTCCCCCAGGAATG	6409-6428		(*22*)
GV132R**	CCRGCRAAGAAAGCTCCAGCCAT	6681-6703	ORF3 (1721 bp)	(*6*)
NoVGII-17F3	CGGCCCGACAAAACTGAG	4656-4673	ORF1	This paper
NoVGII-17R3	CTACTTGAGGGTGGCGTGACAT	7312-7333	ORF3	This paper
NGII17SeqF11	TGACGCCGCTCCATCTAA	5076-5093	ORF2	This paper
NGII17SeqF12	AACAGCCGCATGAGATTAGTGG	5575-5596		This paper

NGII17SeqF13	TCCAGCCGACAAAATTCACACCAG	6200-6223	This paper
NGII17SeqF16	CCCCATCGATTCGCTTTTCACC	5790-5811	This paper
NGII17SeqR12	TGGCACATCATCAGTTGGGTCAT	5993-6015	This paper
NGII17SeqR13	CGGGTGGCCATCATCGTCAT	6236-6255	This paper
NGII17SeqR14	CCTTCGCCCATTCCCAGTTCC	6649-6669	This paper
NGII17SeqR16	CTAGGCTTTGCAGGTTTTGATTG	7080-7102	This paper

*****Locations of oligonucleotides are relative to norovirus GII/Hu/US/2014/GII.17/Gaithersburg (Genbank accession number KR083017).

**Primer GV132R is paired with COG2F to generate the full-length VP1 sequence.
